# Laboratory Monitoring of Patients Treated with Antihypertensive Drugs and Newly Exposed to Non Steroidal Anti-Inflammatory Drugs: A Cohort Study

**DOI:** 10.1371/journal.pone.0034187

**Published:** 2012-03-27

**Authors:** Jean-Pascal Fournier, Maryse Lapeyre-Mestre, Agnès Sommet, Julie Dupouy, Jean-Christophe Poutrain, Jean-Louis Montastruc

**Affiliations:** 1 Laboratoire de Pharmacologie Médicale et Clinique, Faculté de Médecine, Université Paul Sabatier, Toulouse, France; 2 UMR1027, Inserm, Toulouse, France; 3 Département Universitaire de Médecine Générale, Faculté de Médecine, Université Paul Sabatier, Toulouse, France; 4 Service de Pharmacologie Clinique, Centre Midi-Pyrénées de Pharmacovigilance, de Pharmacoépidémiologie et d'Information sur le Médicament, Centre Hospitalier Universitaire de Toulouse, Toulouse, France; University of Sao Paulo Medical School, Brazil

## Abstract

**Background:**

Drug-Drug Interactions between Non Steroidal Anti-Inflammatory Drugs (NSAIDs) and Angiotensin Converting Enzyme Inhibitors (ACEIs), Angiotensin Receptor Blocker (ARBs) or diuretics can lead to renal failure and hyperkalemia. Thus, monitoring of serum creatinine and potassium is recommended when a first dispensing of NSAID occur in patients treated with these drugs.

**Methods:**

We conducted a pharmacoepidemiological retrospective cohort study using data from the French Health Insurance Reimbursement Database to evaluate the proportion of serum creatinine and potassium laboratory monitoring in patients treated with ACEI, ARB or diuretic and receiving a first dispensing of NSAID. We described the first dispensing of NSAID among 3,500 patients of a 4-year cohort (6,633 patients treated with antihypertensive drugs) and analyzed serum creatinine and potassium laboratory monitoring within the 3 weeks after the first NSAID dispensing.

**Results:**

General Practitioners were the most frequent prescribers of NSAIDs (85.5%, 95% CI: 84.3–86.6). The more commonly prescribed NSAIDs were ibuprofen (20%), ketoprofen (15%), diclofenac (15%) and piroxicam (12%). Serum creatinine and potassium monitoring was 10.7% (95% CI: 9.5–11.8) in patients treated by ACEIs, ARBs or diuretics. Overall, monitoring was more frequently performed to women aged over 60, treated with digoxin or glucose lowering drugs, but not to patients treated with ACEIs, ARBs or diuretics. Monitoring was more frequent when NSAIDs' prescribers were cardiologists or anesthesiologists.

**Conclusion:**

Monitoring of serum creatinine and potassium of patients treated with ACEIs, ARBs or diuretics and receiving a first NSAID dispensing is insufficiently performed and needs to be reinforced through specific interventions.

## Introduction

Because Non Steroidal Anti-Inflammatory Drugs (NSAIDs) inhibit cyclooxigenase enzymes (COX) and prevent prostaglandin synthesis, their drug-drug interactions (DDIs) with antihypertensive drugs can lead to adverse drug reactions [1]. NSAIDs association with these drugs can increase arterial blood pressure. Concomitant use of NSAIDs with Angiotensin Conversion Enzyme inhibitors (ACEIs), Angiotensin Receptors Blockers (ARBs) or diuretics can also precipitate acute renal failure, hyponatremia or hyperkalemia, especially when used on elderly or dehydrated individuals. Moreover, the risk of significant renal impairment is associated with the number of these drugs, when they are associated [2].

In France, two drug interaction compendia are available. The main one is provided by the *Agence Française de Sécurité Sanitaire et des Produits de Santé* (*Afssaps*, the French Drug Agency) and is available online [3]. The concise information provided in this guideline is used by the main drug databases (especially the French National Formulary: *Vidal*® [4]). The second one is the annual supplement of the French independent drug information bulletin *La Revue Prescrire* [5]. Recommendations are to monitor serum creatinine alone [4], and even serum creatinine and potassium [5] whenever NSAIDs are first prescribed with ACEIs, ARBs or diuretics (Table S1). However, prescribers' compliance to these recommendations had not been fully evaluated. Thus, we performed a pharmacoepidemiological cohort study investigating if these laboratory monitoring are currently followed by practitioners.

## Methods

All the French population is covered by a publicly funded health system. The French Health Insurance Reimbursement Database gathers information concerning these patients. Four kinds of data are computerized in this database: demographic characteristics of users, characteristics of health professionals, data concerning health facilities and reimbursement data (drug, laboratory, radiology, medical acts) [6]. Concerning drug dispensing, the database contains information on the date of dispensing, quantity dispensed, and prescriber. Drugs are classified according to the Anatomical Therapeutic Chemical system [7].

### Study population

We extracted a random sample of patients (sample rate: 5%, as provided by the French Health Insurance System Database), living in the Midi-Pyrénées area (2,600,000 inhabitants) between 1 April 2005 and 1 April 2006, receiving at least two prescriptions of the same antihypertensive drug and not receiving any NSAID (including topical, injectable and oral forms) during this period. Inclusion in the study was on 1 April 2006 for all patients and the maximal follow-up was 4 years (until 31 March 2010, because of database size limitations). Patients were considered lost-to-follow-up if having no drug dispensing for more than 3 months. All data were anonymous in conformity with the French Law of Privacy (8).

The following oral and injectable NSAIDS marketed in France during the period of study were extracted: arylcarboxylic acids (aceclofenac, alminoprofen, diclofenac, etodolac, fenoprofen, flurbiprofen, ibuprofen, ketoprofen, naproxen, nabumetone, tiaprofenic acid), oxicams (meloxicam, piroxicam, tenoxicam), coxibs (celecoxib), acetylsalicylic acid (excluding anti-platelet doses) and others (indometacin, sulindac, phenylbutazone, nimesulide, mefenamic acid, morniflumate, niflumic acid).

Antihypertensive drugs included beta-blocking agents, ACEI, ARBs, diuretics (except eplerenone), calcium channel blockers (except bepridil), alpha-blocking agents or other drugs (centrally acting antihypertensive drugs, minoxidil and dihydralazine). Renin inhibitors were not available in France during the period of study and thus were not included in the analysis. We took into account fixed combinations of antihypertensive drugs as separate drugs. The level of renal failure and hyperkalemia risk caused by NSAID/antihypertensive DDIs was graduated in risk levels, as showed in table 1.

**Table 1:** Risk of renal failure/hyperkalemia caused by DDIs with NSAIDs according to classes of antihypertensive drugs.

Risk group	Antihypertensive classes
No risk	Beta-blocking agents
	Calcium channel blockers
	Alpha-Blocking agents
	Other antihypertensives
At risk of DDI‡	
«one drug»	Diuretics or ACEI or ARB
«two drugs and more»	Diuretics + ACEI/ARB

DDI, Drug-Drug Interaction; ACEIs, angiotensin converting enzyme inhibitors; ARBs, angiotensin receptor blockers.

‡ At risk of renal failure/hyperkalemia caused by Drug-Drug Interactions (DDIs) between NSAIDs and antihypertensives.

For chronically used drugs, data were extracted between 6 months before and 6 months after the first NSAID dispensing. Patients were considered exposed between the first and the last dispensing of these drugs in this one-year time frame.

Laboratory monitoring of serum creatinine and potassium were considered relevant if occurring within 3 weeks after the start of NSAID. This time frame is pharmacologically relevant and already used in monitoring of initiation or intensification of Renin Angiotensin System Inhibitors (RASIs) [8]. We also explored laboratory monitoring before start of NSAID with monitoring occurring in the year previous first prescription of NSAID.

### Ethics/consent

We performed an observational study on anonymous data. Thus, considering the French legislation, it does not need to be approved by an ethic committee.

### Statistic analysis

Descriptive statistics were computed to characterize patients, drug dispensing, and laboratory monitoring. Factors associated with laboratory monitoring in univariate analysis (p<0.2) were assessed using logistic regression modeling. Characteristics considered in univariate models included age, gender, level of renal failure and hyperkalemia risk caused by DDIs with antihypertensive drugs, digoxin, potassium supplements, glucose lowering drugs, platelet anti-aggregating agents, hospitalizations in the three months previous first dispensing of NSAIDs. Statistical analyses were performed using Stata®, version 11.0 (StataCorp LP, College Station, TX).

## Results

### Cohort

**Figure 1:**
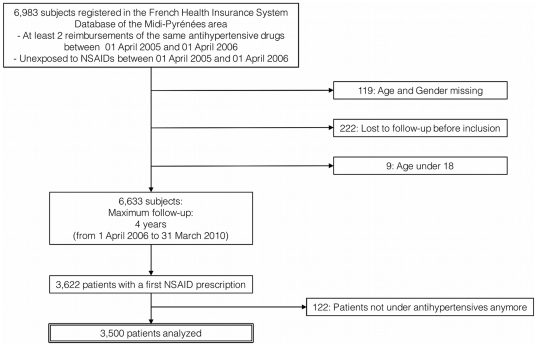
Selection of the 3,500 antihypertensive-treated patients with a first dispensing of NSAIDS.

Over the 6,633 patients of the cohort, 3,622 had a first dispensing of NSAIDs during the follow-up (incidence rate: 25.1/100 PY). Among them, 122 were not treated with antihypertensive drug anymore when the first dispensing of NSAIDs occurred and were thus excluded from the analysis (figure 1). Among the 3,500 remaining patients, 22 had a first dispensing of two different NSAIDs, always prescribed by the same physician. The characteristics of the first dispensing of NSAIDs are shown in table 2. Overall, 2,696 (77.0%) patients were classified at risk of DDI («one drug»: 41.6%, «two drugs»: 35.3%). General Practitioners (GPs) prescribed the majority of NSAIDs (85.5%, 95% CI: 84.3–86.6), and mainly ibuprofen (18.6%), diclofenac (16.3%), ketoprofen (15.4%) or piroxicam (13.4%). Dentists preferentially prescribed ibuprofen (52.2%) and surgeons mainly prescribed ketoprofen (15.2%). Cardiologists and anesthesiologists mainly prescribed flurbiprofen (76.9% and 90.5% of their NSAIDs prescriptions).

**Table 2:** Characteristics of the first dispensing of NSAID.

Characteristics	NSAID (all)	Ibuprofen	Ketoprofen	Diclofenac	Piroxicam	Celecoxib	Acetylsalicylic Acid
Number of patients, n (%)	3,500 (100.0)	722 (20.6)	531 (15.2)	526 (15.0)	416 (11.9)	124 (3.5)	144 (4.1)
DDD dispensed, n (m ± sd)	13.5±7.8	7.5±3.9	18.4±8.1	17.3±6.2	13.8±5.2	28.1±6.9	5.8±3.0
Time since index date,d (med [IQR])	376 [170–718]	418 [208–786]	404 [174–828]	444 [194–787]	258 [128–480]	476 [176–833]	321 [169–622]
Prescriber, n (%)							
General Practitioner	2.992 (85.5)	560 (77.6)	464 (87.4)	492 (93.5)	403 (96.9)	116 (93.5)	139 (96.5)
Dentist	274 (7.8)	143 (19.8)	12 (2.3)	4 (0.8)	1 (0.2)	0 (0.0)	1 (0.7)
Other prescribers	230 (6.6)	18 (2.5)	55 (10.3)	30 (5.7)	12 (2.9)	8 (6.4)	4 (2.8)
Surgeons	76 (2.2)	11 (1.5)	31 (5.8)	13 (2.5)	0 (0.0)	0 (0.0)	0 (0.0)
Rheumatologist	57 (1.6)	2 (0.3)	12 (2.3)	9 (1.7)	6 (1.4)	6 (4.8)	1 (0.7)
Anesthesiologist	21 (0.6)	0 (0.0)	1 (0.2)	0 (0.0)	0 (0.0)	0 (0.0)	0 (0.0)
Cardiologist	13 (0.4)	0 (0.0)	0 (0.0)	0 (0.0)	0 (0.0)	0 (0.0)	2 (1.4)
Other	67 (1.9)	5 (0.7)	10 (1.9)	8 (1.5)	6 (1.4)	0 (0.0)	0 (0.0)
Missing	4 (0.1)	1 (0.1)	1 (0.2)	0 (0.0)	0 (0.0)	2 (1.6)	1 (0.7)
Antihypertensive drug DDI risk, n (%)							
No risk	804 (23.0)	168 (23.3)	122 (23.0)	115 (21.9)	95 (22.8)	24 (19.3)	33 (22.9)
At risk of DDI‡	2,696 (77.0)	554 (76.7)	409 (77.0)	411 (78.1)	321 (77.2)	100 (80.6)	111 (77.1)
«one drug»	1,460 (41.7)	289 (40.0)	219 (41.2)	214 (40.7)	190 (45.7)	57 (46.0)	59 (39.6)
«two drugs and more »	1,236 (35.3)	265 (36.7)	190 (35.8)	197 (37.4)	131 (31.5)	43 (34.7)	54 (37.5)

n, number; m, mean; sd, standard deviation; d, days; med, median; IQR, interquartile range; DDI Drug-Drug interaction.

NSAIDs, non steroidal anti-inflammatory drugs; DDD, defined Daily Dose;

Acetylsalicylic Acid: excluding anti-platelet dose.

‡ At risk of renal failure/hyperkalemia caused by Drug-Drug Interactions (DDIs) between NSAIDs and antihypertensives (see table 1).

### Serum creatinine and potassium monitoring

Baseline complete monitoring was performed in 59.1% (95% CI: 57.3–61.0) of patients treated by ACEIs, ARBs or diuretics (“at risk” group). In only 10.7% (95% CI: 9.5–11.8) of these patients, a complete laboratory monitoring was recorded in the three weeks after NSAID initiation. This monitoring occurred by mean on the 8^th^ day (8.5±6.1) after the first dispensing of NSAID. Table 3 shows the different rates of monitoring according to DDIs risks.

**Table 3:** Serum creatinine and potassium monitoring before* and after start of NSAID.†

Risk group, n	Laboratory monitoring before start of NSAID* [n (%)]			Laboratory monitoring after start of NSAID† [n (%)]		
	Creatinine and potassium monitoring	Creatinine monitoring	Potassium monitoring	Creatinine and potassium monitoring	Creatinine monitoring	Potassium monitoring
	(n = 1.942)	(n = 534)	(n = 374)	(n = 347)	(n = 532)	(n = 374)
No risk, 804	348 (43.3)	511 (63.6)	367 (45.6)	59 (7.3)	109 (13.6)	66 (8.2)
At risk of DDI‡, 2,696						
«one drug», 1,460	833 (57.0)	1,038 (71.1)	870 (59.6)	154 (10.5)	225 (15.4)	164 (11.2)
«two drugs and more», 1,236	761 (61.6)	880 (71.2)	792 (64.1)	134 (10.8)	198 (16.0)	142 (11.5)

NSAIDs, non steroidal anti-inflammatory drugs.

* in the year previous first NSAID dispensing.

† in the 3 weeks after start of NSAID.

‡ At risk of renal failure/hyperkalemia caused by Drug-Drug Interactions (DDIs) between NSAIDs and antihypertensives (see table 1).

Univariate analysis showed that all the characteristics selected were associated to serum creatinine and potassium monitoring (table 4). Multivariate analysis performed with these variables (table 5), showed that being aged over 60 (60–70: OR = 1.78, 95% CI: 1.00–3.15; 70–80: OR = 2.17, 95% CI: 1.26–3.73; over 80: OR = 3.14, 95% CI: 1.79–5.48) and a woman (OR = 1.26, 95% CI: 1.00–1.59) was associated with a more frequent monitoring. Monitoring was also more frequent among patients treated with potassium supplements (OR = 2.47, 95% CI: 1.49–4.12) and glucose lowering drugs (OR = 1.58, 95% CI: 1.22–2.06) but not in patients treated with ACEI, ARBs or diuretics («one drug» OR = 1.27, 95% CI: 0.92–1.75; «two drugs» OR = 1.28, 95% CI: 0.92–1.77). Monitoring was more frequent when the prescriber of NSAID was a cardiologist or an anesthesiologist (OR = 3.32, 95% CI: 1.53–7.26).

**Table 4:** Serum creatinine and potassium monitoring after start of NSAID according to NSAID prescriber.

NSAID Prescriber (n)	Laboratory monitoring after start of NSAID* [n (%)]		
	Creatinine and potassium monitoring	Creatinine monitoring	Potassium monitoring
	(n = 347)	(n = 532)	(n = 372)
General Practitioner (2,992)	294 (9.8)	459 (15.3)	317 (10.6)
Dentist (274)	24 (8.8)	35 (12.8)	24 (8.8)
Other prescribers (238)	29 (12.2)	38 (16.0)	31 (13.0)
Anesthesiologist (21)	4 (19.0)	7 (33.3)	5 (23.8)
Cardiologist (13)	5 (38.5)	5 (38.5)	5 (38.5)
Surgeons (76)	9 (11.8)	14 (18.4)	9 (11.8)
Rheumatologist (56)	3 (5.4)	4 (7.1)	3 (5.4)
Other (67)	8 (12.5)	8 (12.5)	9 (14.1)

NSAIDs, non steroidal anti-inflammatory drugs.

**Table 5:** Factors associated with serum creatinine and potassium after start of NSAID.

Factors	Unadjusted odds ratio	p	Adjusted odds ratio*	p
	[95% confidence interval]		[95% confidence interval]	
Age, years (<50 years as reference)				
50–60	1.70 [0.94–3.07]	0.075	1.64 [0.91–2.95]	0.147
60–70	1.89 [1.07–3.34]	0.028	1.78 [1.00–3.15]	0.049
70–80	2.51 [1.44–4.36]	0.001	2.31 [1.32–4.04]	0.003
>80	3.42 [1.94–6.03]	0.000	3.36 [1.90–5.96]	0.000
Gender (male as reference)	1.18 [0.95–1.48]	0.129	1.26 [1.00–1.59]	0.044
Concomitant drugs†				
Potassium supplements	2.88 [1.74–4.75]	0.000	2.47 [1.49–4.12]	0.001
Glucose lowering drugs	1.58 [1.22–2.04]	0.001	1.58 [1.22–2.06]	0.001
Digoxin	2.04 [1.17–3.54]	0.012	1.60 [0.91–2.82]	0.106
Platelet aggregation inhibitors	1.49 [1.17–1.88]	0.001	1.20 [0.93–1.54]	0.153
Risk level of DDI‡ (no risk as reference)				
One drug	1.49 [1.09–2.04]	0.013	1.27 [0.92–1.75]	0.139
Two drugs	1.53 [1.11–2.11]	0.009	1.28 [0.92–1.77]	0.140
Hospitalizations§ (any)	2.68 [1.14–6.27]	0.023	2.09 [0.87–5.00]	0.097
NSAID prescriber				
Cardiologist or Anesthesiologist¶	3.33 [1.54–7.19]	0.002	3.32 [1.53–7.26]	0.003

* Adjusted for age, gender, exposure to potassium supplements, glucose lowering drugs and NSAIDs prescriber.

† according to ATC classification.

‡ Risk level of renal failure/hyperkalemia caused by Drug-Drug Interactions (DDIs) between NSAIDs and antihypertensives (see table 1).

§ in the 6 months before inclusion.

¶ Compared to other prescribers.

## Discussion

Our study was performed to evaluate the implementation of laboratory monitoring in patients treated with ACEI/ARB/Diuretic plus NSAID. Our results show that despite well-known potential biochemical disturbances, serum creatinine and potassium monitoring were recorded in less than 11% of patients at risk. Monitoring occurred at day 8 and was more frequently performed to women aged over 60, treated with potassium supplements or glucose lowering drugs, but not to patients treated with ACEI, ARBs or diuretics. Monitoring was more frequent when NSAIDs' prescriber was a cardiologist or anesthesiologist.

The characteristics of NSAID prescriptions are close to the ones found in a monthly prevalence descriptive study leaded in the same area in 2006 [9]. First prescriptions of NSAIDs among hypertensive patients do not differ from NSAIDs prescriptions in general population, as the more frequently prescribed NSAIDs are in both studies ibuprofen, ketoprofen, diclofenac and piroxicam. Our study adds information on specificities of NSAIDs' prescriptions among prescribers. Among GPs, the rate of piroxicam prescriptions remains high, but is decreasing compared to previous studies performed in the same area [10]. This phenomenon may reflect the recent recommendation from the *Haute Autorité de Santé* (equivalent of the National Institute for Health and Clinical Excellence in France), underlining that piroxicam remains a second-line NSAID [11] in its main indications. We also found that dentists' prescriptions were preferentially ibuprofen, the NSAID commonly prescribed for its anti-inflammatory and analgesic effect in acute dental pain [12]. This information is reassuring, as low dose ibuprofen is believed to be (with naproxen) the least harmful NSAID regarding cardiovascular events [13].

To our knowledge, this study is the first one describing monitoring of serum creatinine and potassium in patients at risk of renal failure or hyperkalemia caused by NSAID DDIs with ACEI, ARBs or diuretics. The rate found in our study (around 11%) is unsurprisingly low. Low monitoring rates have been found in previous study whether in RASIs initiation (34% of control in the first 3 weeks [8]) or with chronically prescribed ARBs/ACEIs/diuretics (68 to 72% of annual control [14]). Furthermore, in our study the level of DDIs risk is not associated with a greater control. In Bootsma *et al.* study [8], being under NSAIDs was not associated to an adequate control either in patients starting ACEI/ARB therapy.

This study underlines the important lack of implementation of guidelines for DDIs between NSAIDs and antihypertensives. This finding is quite ambiguous, as GP have previously reported their concerns about NSAIDs safety of use in daily practice and claimed a caution approach in NSAID prescription [15]. As an explanation to this phenomenon, two approaches can be considered focusing on guideline-related factors and GPs-related factors [16]. Concerning the quality of the interaction compedia, one should underline that the main one, provided by the French Drug Agency, is available online [3]. The concise information provided in this guideline is used by the main drug databases (especially the French National Formulary: *Vidal*® [4]) and thus in the main medical software, which automated prompts and alerts have already demonstrated positive effects on decreasing preventable adverse drug events [17]. The main limitation of the recommendations could be the absence of explicit time frame in which the monitoring should be performed. The impact of this lack of precision remains uncertain. Moreover, the recommendations are different in other compendia. Surprisingly, the *British National Formulary* emphasizes on the increased risk of nephrotoxicity of the association between NSAIDs and ACEIs/ARBs/diuretics, [18] but does not provide recommendations of laboratory monitoring. This lack of consistency between drug interaction compendia has already been raised [19] and underlines the necessity for their standardization.

Regarding GPs-related factors for the non-implementation of drug prescribing guidelines, GPs may consider guidelines as too stringent in general. They consider laboratory monitoring as time-consuming, especially when they are uncertain that monitoring was already performed by another provider [20]. GPs also raise concerns about the real impact of computerized clinical decision support to increase implementation of guidelines, as a phenomenon of alert fatigue could occur. Weingart *et al.* [21] recently emphasized on the necessity for computerized alerts to be adapted to clinicians.

In the present study, cardiologists and anesthesiologists prescribed more frequently adequate monitoring. This phenomenon can be explained by an increased prescription of flurbiprofen within these two medical specialties. Flurbiprofen is marketed in France for prevention of reinfarction and reocclusion after successful thrombolysis or angioplasty in acute myocardial infarction, in patients for whom aspirin is not recommended [22]. Thus, these patients could have more frequent monitoring because of their condition. Another explanation could be that these medical specialties are more aware of the risk evaluated in the present study.

### Limitations

The use of the French Health Insurance Reimbursement Database in pharmacoepidemiological studies has already been fully described [6], but it implies some limitations. As for many administrative databases, we did not have access to medical characteristics of the patients. This involves using medications as proxies of morbidities (e.g. glucose lowering drugs for diabetes mellitus [23]). We were not able to extract some characteristics associated to serum creatinine and potassium monitoring in a previous study [24], because of database limitations. In this study of Raebel *et al.*, increasing number of outpatient visits and diagnoses of chronic heart failure or kidney disease were associated to annual monitoring. Furthermore, the disease necessitating NSAID prescription could alone be a condition implying a monitoring of serum creatinine and potassium (e.g. renal colic [25]). Moreover, the database only records monitoring that have been performed and not all the ones that have been prescribed. A lot of patients-related situations (reluctance to blood test, doctor shopping, excessive self-confidence towards adverse drug reactions…) could have an impact on the realization of monitoring in a reasonable time frame.

Finally, the low prevalence of complete monitoring could have been underestimated. In our study, we only have access to ambulatory biochemical monitoring and thus could have missed the ones realized during hospitalizations. On the other hand, one could have underestimated the prevalence of ibuprofen and aspirin consumption, as these specific NSAIDs can be sold out-of-the-counter and thus not recorded in the French Health Insurance Reimbursement Database.

### Conclusion

The low prevalence of serum creatinine and potassium monitoring shows a very poor implementation of guidelines. Further studies are required to correlate this low prevalence with a potential increased risk of severe adverse drug reactions. Moreover, intervention studies are required to improve the knowledge of this specific risk, especially among GPs.

## Supporting Information

Table S1 **Drug interactions between NSAIDs and antihypertensive drugs according to l'Agence Française de Sécurité Sanitaire et des Produits de Santé ( = French Drug Agency) and La Revue Prescrire.** † There are four levels of seriousness, based on the clinical management which is recommended: ‘contraindication’ (absolute), ‘avoid’ (relative contraindication), ‘precaution for use’ (combination possible if recommendations are followed), and ‘to take into account’ (no specific recommendation) * Afssaps: Agence Française de Sécurité Sanitaire et des Produits de Santé ( = French Drug Agency).
(DOCX)
