# Supplementary material for: Laboratory Monitoring of Patients Treated with Antihypertensive Drugs and Newly Exposed to Non Steroidal Anti-Inflammatory Drugs: A Cohort Study
Source: PLoS One. 2012 Mar 27;7(3):e34187. doi: 10.1371/journal.pone.0034187 (PMC3313991; doi:10.1371/journal.pone.0034187)
Supplement: Table S1 — Drug interactions between NSAIDs and antihypertensive drugs according to l'Agence Française de Sécurité Sanitaire et des Produits de Santé ( = French Drug Agency) and La Revue Prescrire. † There are four levels of seriousness, based on the clinical management which is recommended: ‘contraindication’ (absolute), ‘avoid’ (relative contraindication), ‘precaution for use’ (combination possible if recommendations are followed), and ‘to take into account’ (no specific recommendation) * Afssaps: Agence Française de Sécurité Sanitaire et des Produits de Santé ( = French Drug Agency). (DOCX) [file pone.0034187.s001.docx]

|  | Afssaps* | | Revue Prescrire | |
| --- | --- | --- | --- | --- |
|  | Adverse reaction | Level of seriousness^†^ and recommendations | Adverse reaction | Level of seriousness and recommendations |
| **Beta-blocking agents** |  |  |  |  |
| Acebutolol, atenolol, betaxolol, bisoprolol, carteolol, carvedilol, celiprolol, labetalol, metoprolol, nadolol, nebivolol, oxprenolol, penbutolol, pindolol, propranolol, sotalol, tertatolol, timolol | Inhibition of antihypertensive effect | **To take into account** | Inhibition of antihypertensive effect |  |
| **Angiotensin Converter Enzyme Inhibitors** |  |  |  |  |
| Benazepril, captopril, cilazapril, enalapril, fosinopril, imidapril, lisinopril, moexipril, perindopril, quinapril, ramipril, trandolapril, zofenopril | Inhibition of antihypertensive effect Acute renal failure in "at risk" patient (elderly or dehydration) | **Precaution for use**  Hydrate the patient et monitor renal function after onset of therapy | Inhibition of antihypertensive effect Acute renal failure in patients with hypovolemia or dehydration  Hyperkaliemia Hyponatremia | **To avoid**  Monitor creatinine clearance and serum potassium |
| **Angiotensin Receptor Blockers** |  |  |  |  |
| Candesartan, eprosartan, irbesartan, losartan, olmesartan, telmisartan, valsartan | Inhibition of antihypertensive effect Acute renal failure in "at risk" patient (elderly or dehydration) | **Precaution for use**  Hydrate the patient et monitor renal function after onset of therapy | Inhibition of antihypertensive effect Acute renal failure in patients with hypovolemia or dehydration  Hyperkaliemia Hyponatremia | **To avoid** Monitor creatinine clearance and serum potassium |
| **Renin inhibitors** |  |  |  |  |
| Aliskiren |  |  | Inhibition of antihypertensive effect Risk of acute renal failure | **To avoid** Monitor creatinine clearance and serum potassium |
| **Calcium channel blockers** |  |  |  |  |
| Amlodipine, diltiazem, felodipine, isradipine, lacidipine, lercanidipine, manidipine, mibefradil, nicardipine, nifedipine, nitrendipine, verapamil |  |  | Inhibition of antihypertensive effect |  |
| **Diuretics** |  |  |  |  |
| *Low ceiling diuretics (including thiazides):* altizide, bendroflumethiazide, chlortalidone, cicletanine, clopamide, cyclothiazide, hydrochlorothiazide, indapamide, methylclothiazide, xipamide  *Loop diuretics:* bumetanide, furosemide, piretanide  *Potassium-sparing agents:* spironolactone, amiloride, triamterene | Inhibition of antihypertensive effect Acute renal failure in "at risk" patient (elderly or dehydration) | **Precaution for use**  Hydrate the patient et monitor renal function after onset of therapy | Inhibition of antihypertensive effect Acute renal failure in patients with hypovolemia or dehydration  Hyperkaliemia Hyponatremia | **To avoid** Monitor creatinine clearance and kaliemia |
| **Alpha-Blocking agents** |  |  |  |  |
| Prazosine, urapidil |  |  | Inhibition of antihypertensive effect |  |
| **Other antihypertensives** |  |  |  |  |
| *Centrally acting agents:* clonidine, guanfacine, methyldopa, moxonidine, reserpine, rilmenidine  *Agents acting on arteriolar smooth muscle:* minoxidil, dihydralazine |  |  | Inhibition of antihypertensive effect Risk of dose dependant adverse reaction with NSAID for clonidine et mixonidine | Monitor creatinine clearance |
